# Investigating the role of the HLA-Cw*06 and *HLA-DRB1* genes in susceptibility to psoriatic arthritis: comparison with psoriasis and undifferentiated inflammatory arthritis

**DOI:** 10.1136/ard.2007.071399

**Published:** 2007-08-29

**Authors:** P Y P C Ho, A Barton, J Worthington, D Plant, C E M Griffiths, H S Young, P Bradburn, W Thomson, A J Silman, I N Bruce

**Affiliations:** 1 arc-Epidemiology Unit, Stopford Building, University of Manchester, Manchester, UK; 2 Dermatology Centre, Hope Hospital, University of Manchester, Manchester, UK; 3 Department of Public Health and Epidemiology, University of Birmingham, UK

## Abstract

**Objective:**

Psoriasis of early onset (type I; age of onset ⩽40 years) is associated with HLA-Cw*06 while the shared epitope (SE) is associated with rheumatoid arthritis susceptibility. Our aim was to investigate the role of HLA-Cw*06 and *HLA-DRB1* genes (including SE) with psoriatic arthritis (PsA) susceptibility.

**Methods:**

In a case–control association study, HLA-Cw*06 phenotype frequencies were compared between patients with PsA (n = 480), psoriasis alone (n = 611) and healthy controls (n = 166). Similarly, at the *HLA-DRB1* locus, phenotype and SE frequencies were compared in patients with PsA (n = 480), early undifferentiated inflammatory arthritis alone (n = 1621) and healthy controls (n = 537).

**Results:**

The HLA-Cw*06 phenotype was associated with type I psoriasis (OR 6.9, 95% CI 4.4, 11.1, p = 2.2×10^−21^) and with patients with PsA having type I psoriasis (OR 5.0, 95% CI 3.2, 7.9, p = 4.39×10^−13^), but not with patients with PsA having type II psoriasis (age of onset >40 years). HLA-DRB1*07, in linkage disequilibrium with HLA-Cw*06, was also associated with patients with PsA having type I psoriasis (OR 2.7, 95% CI 2.1, 3.7, p<0.00001). HLA-DRB1*04 alleles and the SE were associated with undifferentiated inflammatory arthritis but not with PsA.

**Conclusions:**

The SE is not a PsA susceptibility locus. HLA-Cw*06 and HLA-DRB1*07 are associated with patients with PsA having type I psoriasis, suggesting that the primary association is with age of onset of psoriasis. Patients with PsA having type I psoriasis, therefore, have a genetic background different to those with type II psoriasis, and adjustment for this is necessary in future studies that investigate the genetic susceptibility of PsA.

Psoriatic arthritis (PsA) is commonly defined as “an inflammatory arthritis (IA) associated with psoriasis, which is usually negative for rheumatoid factor (RF)”.^1^ A strong genetic component to susceptibility is suggested by the sibling recurrence risk ratio (λs), which is estimated to be 27.^2^ ^3^ Part of the genetic predisposition is likely to be explained by genes within the major histocompatibility complex (MHC) region. For example, human leucocyte antigen (HLA) associations with psoriasis and IA in the form of rheumatoid arthritis (RA) are well characterised and, in each case, the MHC genes involved are recognised as the major disease susceptibility locus. Psoriasis has two distinct ages of onset: type I, early onset disease occurring at ⩽40 years of age and type II, late onset occurring at >40 years of age. Carriage of the HLA class I allele, HLA-Cw*06 is associated with type I but not type II psoriasis.^4^^–^13 In contrast, RA is associated with carriage of the shared epitope (SE) of the HLA class II *DRB1* gene (a group of *DRB1* alleles sharing a conserved amino acid motif in the third hypervariable region of the DRβ chain).^14^ HLA-Cw*06 is not found on haplotypes encoding the SE.

For PsA disease susceptibility, separating the HLA-related genetic contribution from the contribution to psoriasis and IA alone is a challenge. There is evidence to suggest that carriage of the HLA-Cw*06 allele is associated with patients with PsA with type I psoriasis.^12^ ^15^^–^19 Studies of the HLA class II *DRB1* gene have reported that the HLA-DRB1*07 phenotype is associated with peripheral arthritis in PsA while HLA-DRB1*04 is associated with a subgroup of patients with PsA with polyarthritis mimicking that of RA.^20^^–^26 In one study of the *HLA-DRB1* locus, HLA-DRB1*0402 (which is not a SE allele) was found to occur more frequently in patients with PsA compared with RA or healthy controls, whereas HLA-DRB*0401 (which is a SE allele) occurred less frequently.^27^ Conflicting results have been reported with regard to the HLA-DRB1*02 (HLA-DRB*15 or HLA-DRB*16) and PsA susceptibility with a decrease found in a UK study, but not in a cohort from Toronto.^28^ ^29^ More recently, a UK study showed no overall difference in the frequency of the SE between PsA cases and controls.^28^

Of the associations reported, however, it is still unclear whether the primary association is with PsA itself or secondary to one of the two constituent components, ie, IA or psoriasis. In previous studies, all appropriate comparison groups have not been analysed and some studies have also lacked power to address this question. The aim of this study was to compare the association between HLA-Cw*06 and *HLA-DRB1*, including the SE, with PsA susceptibility by comparing these phenotypes in patients with PsA, early undifferentiated inflammatory arthritis (UIA) alone, psoriasis alone and healthy controls.

## METHODS

### Overview

For this study, we did not investigate HLA-Cw*06 carriage in UIA patients or *HLA-DRB1* allele carriage in patients with psoriasis as previous studies have excluded a role for these genes in the respective conditions.^12^ ^30^^–^33 A case–control association study was performed to investigate the role of HLA-Cw*06 in determining susceptibility to PsA by comparing allele and phenotype frequencies between patients with PsA, psoriasis alone and healthy controls. For the *HLA-DRB1* gene variants, HLA-DRB1*04, HLA-DRB1*07, HLA-DRB1*02 (HLA-DRB*15 or HLA-DRB*16) and SE phenotype frequencies were compared in patients with PsA, UIA alone and healthy controls. Stratification analyses were performed by subdividing patients with PsA into type I and type II psoriasis according to the age at onset of their skin disease (type I = age of onset ⩽40 years and type II = >40 years of age). Analyses were also repeated in subsets of patients with PsA stratified by the presence of RF.

### Subjects

#### Patients with psoriatic arthritis

The recruitment of patients with PsA for this study has been described previously.^34^ In brief, patients with PsA (n = 480) under active follow-up by hospital rheumatologists were recruited from throughout the UK with the majority of them coming from north-west England. All patients satisfied the inclusion criteria of having both clinically documented inflammatory synovitis and psoriasis regardless of their RF status. A trained research nurse interviewed the patients and completed a standardised clinical history and examination protocol. Detailed demographic and clinical information were obtained and whole blood was taken for the measurement of RF status, DNA extraction and subsequent genetic analysis.

#### Patients with psoriasis

As described previously,^35^ patients with type I psoriasis (age of onset ⩽40 years) (n = 611) were recruited via the Dermatology Centre at Hope Hospital in Manchester. Some of the patients with psoriasis may have an IA, but this was not documented for the majority. A subset (n = 229) underwent an examination to exclude inflammatory joint involvement.

#### Undifferentiated inflammatory arthritis

Patients with early UIA were recruited from the Norfolk Arthritis Register (NOAR) as described previously.^36^ This is a primary-care-based inception cohort of subjects with primary UIA. Patients were aged ⩾16 years with two or more inflamed peripheral joints lasting at least 4 weeks. For the purpose of this study, all patients with *HLA-DRB1* data available were included (n = 1621).

#### Population controls

Control subjects without a history of IA or psoriasis were recruited from blood donors and general practice registers (n = 537).

All patients and controls were white Caucasians of British descent. They were recruited with ethical committee approval (MREC 99/8/84 (PsA samples); LREC 00089 (psoriasis samples), LREC 2003-075 (NOAR samples)) and provided written informed consent.

### HLA typing

Both broad HLA genotyping (*HLA-Cw* and *HLA-DR*) and subtyping to define SE alleles were performed using 50 ng of genomic DNA amplified with the Dynal RELI SSO *HLA-Cw* typing and *HLA-DRB1* kits (http://www.dynalbiotech.com) using a third of the specified volumes for the polymerase chain reaction reagents in a 20 μl reaction instead of 60 μl as described previously.^34^  Alleles were assigned using the Pattern Matching Program provided by Dynal (Invitrogen Ltd, Paisley, UK).

### Statistical analysis

Allele and phenotype frequencies for HLA-Cw*06 were compared between PsA cases, psoriasis cases and controls using the χ^2^ test implemented in STATA 8.

For the *HLA-DRB1*gene, HLA-DRB1*04, HLA-DRB1*07, HLA-DRB1*02 (HLA-DRB*15 or HLA-DRB*16) and SE phenotype frequencies were compared between cases with PsA, UIA and controls using the χ^2^ test implemented in STATA 8. The SE was defined by the presence of any of the following alleles: HLA-DRB1*0101, HLA-DRB1*0102, HLA-DRB1*0104, HLA-DRB1*0401, HLA-DRB1*0404, HLA-DRB1*0405, HLA-DRB1*0408 and HLA-DRB1*1001.

The PsA cohort was divided into patients with type I and type II psoriasis by their age at onset of their psoriasis (⩽40 years or >40 years, respectively) and the analyses were repeated for each subset of PsA. Similar analysis was undertaken after stratifying the PsA cohort by their RF status.

### Linkage disequilibrium analysis

Pairwise linkage disequilibrium (LD) measures (both D′ and r^2^) were investigated between HLA-Cw*06 and HLA-DRB1*07 using HelixTree (Golden Helix Inc, Bozenian, MT, USA). These data has been reported previously.^34^ 

## RESULTS

A summary of the samples used for the HLA-Cw*06 and *HLA-DRB1* analysis is provided in table 1.

**Table 1:** Number of subjects in study (where data are available)

Subjects	HLA-Cw*06 information: n	*HLA-DRB1 *information: n
PsA whole cohort	453	465
PsA with type I psoriasis	335	342
PsA with type II psoriasis	115	120
Psoriasis (all type I)	611	NA
UIA	NA	1621
Population controls	166	537

UIA, early undifferentiated inflammatory arthritis; PsA, psoriatic arthritis; NA, not available.

For three PsA cases, data were not available as to the type of psoriasis present.

### Patient characteristics

#### Psoriatic arthritis

The characteristics of the PsA cohort have been described previously.^34^ There was an almost equal gender distribution with 57% being female and 74% having type I psoriasis. The median duration of psoriasis was 19 years (interquartile range (IQR) 9–33) and the median duration of joint disease was 10 years (IQR 5–19). A majority (63%) developed psoriasis before the onset of joint disease. RF was present in 17% (titre >1:40), 81% had nail involvement, 57% had five or more damaged joints (polyarthritis subgroup) and the median HAQ score was 1.25. As shown previously, patients with PsA with type I psoriasis have a stronger family history of both skin and joint disease and tend to develop arthritis after the onset of psoriasis.^19^ ^37^ In addition, patients with PsA with type I psoriasis had a longer duration of joint disease and more nail involvement, but a lower median HAQ score and fewer involved joints compared with those with type II psoriasis.

#### Psoriasis

All patients had type I psoriasis with 46% (283 of 611) being female. The median age of onset of psoriasis was 19 years (IQR 13–27). Some of these patients may have an unrecognised IA, but a subset (n = 229) have been specifically examined by a dermatologist to exclude an IA. All 611 patients with psoriasis were included in the analysis. No significant differences in clinical, demographic or HLA-Cw*06 carriage data were observed between those patients with psoriasis in whom PsA had been specifically excluded and those where it had not (HLA-Cw*06 carriage was 43% in those without PsA versus 40% in the remainder, p = 0.44).

#### Early undifferentiated inflammatory arthritis

Within the UIA cohort, 1053 (65%) were female. At baseline, the median disease duration was 6 months (IQR 3–12); RF was present at a titre >1:40 in 452/1433 (31.5%) and 743 (48.3%) satisfied the American College of Rheumatology criteria for RA.^38^ By year 5, 11% of the patients were recorded to have psoriasis in addition to their IA and by year 10 of follow-up, 12% developed psoriasis.

#### Controls

In the population control cohort, gender information was available for 268 subjects of whom 119 (44%) were female. HLA-Cw*06 data were available for 166, while *HLA-DRB1* data were available for 573 subjects.

#### HLA-Cw*06

The frequency of the HLA-Cw*06 phenotype in the population controls tested in the current study was within the range reported previously^15^ ^16^ ^25^ ^26^ ^39^^–^41 (table 2).

**Table 2:** Comparison of HLA-Cw*06 in PsA cases, psoriasis and controls

HLA-Cw*06	PsA cases			Population controls n = 166	Type I psoriasis n = 611
	Total cohort n = 453	Type I n = 335	Type II n = 115		
0	262 (57.8)	166 (49.6)	94 (81.7)	138 (83.1)	254 (41.6)
1	182 (40.2)	162 (48.4)	19 (16.5)	28 (16.9)	323 (52.9)
2	9 (2)	7 (2)	2 (1.8)	0	34 (5.5)
Phenotype	191 (42.2)	169 (50.4)	21 (18.3)	28 (16.9)	357 (58.4)
p-Value*	5.5×10^−9^	4.39×10^−13^	0.76		2.15×10^−21^

PsA, psoriatic arthritis. Type I, patients with PsA with type I psoriasis; type II, patients with PsA with type II psoriasis.

*Comparison of phenotype (carriage of one or two alleles) with population controls using the χ^2^ test.

Data shown in n (%). Data were not available for three patients as to the type of psoriasis present.

**Figure 1:**
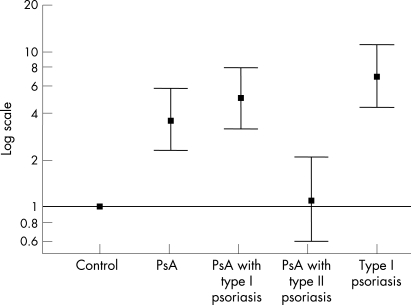
Comparison of HLA-Cw*06 phenotype in psoriatic arthritis (PsA) cases, psoriasis and controls. Odds ratios and 95% confidence intervals are shown on a log scale.

When compared with controls, the HLA-Cw*06 phenotype was shown to be strongly associated with PsA (odds ratio (OR) 3.6, 95% CI 2.3, 5.8 and p = 5.5×10^−9^) (table 2 and fig 1). Stratification analysis in the PsA cohort by RF status made no difference to the result (OR in the RF-negative PsA subgroup 3.6, 95% CI 2.3, 5.9, p = 9.6×10^−9^). However, the association with HLA-Cw*06 was confined to the subgroup of patients with PsA with type I psoriasis (OR 5.0, 95% CI 3.2, 7.9, p = 4.39×10^−13^) and was not observed in patients with PsA with type II psoriasis (OR 1.1, 95% CI 0.6, 2.1, p = 0.76).

For the psoriasis cohort, as expected, HLA-Cw*06 was strongly associated with type I psoriasis compared with controls (OR 6.9 95% CI 4.4 to 11.1, p = 2.15×10^−21^) (table 2 and fig 1). To determine whether HLA-Cw*06 is associated with PsA itself or primarily with psoriasis, we compared the HLA-Cw*06 phenotype frequencies in those patients with PsA with type I psoriasis and patients with type I psoriasis alone. A much weaker association was noted (PsA with type I psoriasis versus type I psoriasis, OR 0.72, 95% CI 0.55, 0.96, p = 0.02), suggesting that the primary association of HLA-Cw*06 is with type I psoriasis and not PsA *per se*.

#### HLA-DRB1

Stratification analysis showed that the HLA-DRB1*07 phenotype was strongly associated in those patients with PsA with type I psoriasis (OR 2.7, 95% CI 2.1, 3.7 and p<0.00001) compared with controls. Although, HLA-DRB1*07 occurred significantly more frequently in PsA cases than controls, we have previously reported that this allele exhibits considerable LD with HLA-Cw*06 (correlation (r^2^) = 0.46).^34^ Therefore, it was not unexpected that, after adjusting for the presence of HLA-Cw*06 phenotype, the association of HLA-DRB1*07 with PsA as a whole group (OR 1.38, 95% CI 0.88, 2.17, p = 0.16) or in the subgroup with type I psoriasis compared with controls (OR 1.63, 95% CI 0.96, 2.78, p = 0.07) was no longer statistically significant. However, the association of PsA with HLA-Cw*06 remained similar after adjusting for the presence of the HLA-DRB1*07 (OR 3.2, 95% CI 2.0 to 5.3), confirming that the primary association is with HLA-Cw*06 and not HLA-DRB1*07.

Patients with PsA negative for RF were less likely to carry the HLA-DRB1*04 phenotype compared with population controls (OR 0.74, 95% CI 0.55, 0.99, p = 0.03), but no difference was observed between those patients with PsA with a positive RF compared with controls (OR 0.96, 95% CI 0.56, 1.61, p = 0.88). In addition, the HLA-DRB1*04 phenotype occurred less frequently in patients with PsA with type I psoriasis compared with population controls (p = 0.004), but no difference was observed in those patients with PsA with type II psoriasis compared with controls (p = 0.45). Within patients with PsA, when the HLA-DRB1*04 phenotype was present, it occurred more commonly in patients with PsA with type II psoriasis compared with those with type I psoriasis (OR 1.81, 95% CI 1.14, 2.86, p = 0.007). No association was detected with those patients with PsA having ⩾5 damaged (poly-damaged) or ⩾5 involved (poly-involved) joints with the HLA-DRB1*04 phenotype compared with population controls (OR 0.87, 95% CI 0.6, 1.2, p = 0.39 and OR 0.81, 95% CI 0.60, 1.08, p = 0.14, respectively).

Table 3 shows that in the UIA cohort, the HLA-DRB1*04 phenotype was more common than population controls (OR 1.40, 95% CI 1.14, 1.72 and p = 0.001), while the HLA-DRB1*07 phenotype was more common in the PsA cohort compared with population controls (OR 2.15, 95% CI 1.62, 2.84, p = 2.6×10^−8^). However, when comparing patients with PsA with type II psoriasis with UIA subjects, no difference was observed between these two cohorts for either the HLA-DRB1*04 or the HLA-DRB1*07 phenotypes (p = 0.35 and p = 0.45 respectively). When compared with patients with PsA with type I psoriasis, the frequency of the HLA-DRB1*04 phenotype was significantly higher in UIA subjects (OR 2.17, 95% CI 1.66, 2.84, p<0.0001). Conversely, the HLA-DRB1*07 phenotype was significantly higher in patients with PsA with type I psoriasis compared with UIA (OR 3.23, 95% CI 2.51, 4.14, p<0.0001).

**Table 3:** *HLA-DRB1* phenotypes in PsA cases, UIA and controls (where data available)

HLA-DRB1	PsA cases			Controls n = 537	p value (PsA whole cohort compared with controls)	UIA n = 1621	p Value (UIA compared with controls)
	Whole cohort n = 465	Type I n = 342	Type II n = 120				
DRB1*02	118 (25)	81 (24)	36 (30)	145 (27)	0.57	342 (21)	0.006
DRB1*04	142 (31)	92 (27)	48 (40)	195 (36)	0.06	719 (44)	0.001
DRB1*07	188 (40)	159 (46)	29 (24)	129 (24)	3.09×10^−8^	344 (21)	0.21

PsA, psoriatic arthritis; UIA, early undifferentiated inflammatory arthritis; type I, patients with PsA with type I psoriasis; type II, patients with PsA with type II psoriasis.

Data shown in n (%) unless stated otherwise.

Previous studies have reported conflicting results with regard to the HLA-DRB1*02 (HLA-DRB*15 or HLA-DRB*16) and PsA susceptibility with a decrease found in a UK study, but not in a cohort from Toronto.^28^ ^29^ We did not observe a difference in our cohort.

### Shared epitope

**Figure 2:**
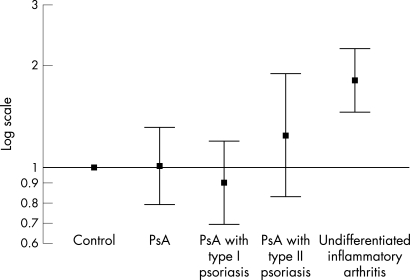
Shared epitope phenotype in psoriatic arthritis (PsA) cases, undifferentiated inflammatory arthritis and controls. Odds ratios and 95% confidence intervals are shown on a log scale.

When comparing patients with PsA and controls, no association was detected with SE allele carriage and PsA (table 4 and fig 2) either in the entire cohort (p = 0.94) or after stratifying the patients with PsA by type I or type II psoriasis or by their RF status. For example, no association was detected when comparing SE phenotype between patients with PsA with type I psoriasis and population controls (OR 0.90, 95% CI 0.68, 1.19, p = 0.43) or when comparing patients with PsA with type II psoriasis with population controls (OR of 1.24, 95% CI 0.82, 1.88, p = 0.28).

**Table 4:** Shared epitope frequency in PsA cases, UIA and controls

Shared epitope	UIA n = 1621	PsA cases			Population controls n = 537
		Whole cohort n = 467	Type I n = 344	Type II n = 120	
0	634 (39.1)	256 (54.8)	197 (57.3)	59 (49.2)	293 (54.6)
1	740 (45.7)	184 (39.4)	126 (36.6)	55 (45.8)	203 (37.8)
2	247 (15.2)	27 (5.8)	21 (6.1)	6 (5.0)	41 (7.6)
Phenotype	987 (60.9)	211 (45.2)	147 (42.7)	61 (50.8)	244 (45.4)
p Value*	3.63×10^−10^	0.94	0.43	0.28	

UIA, early undifferentiated inflammatory arthritis; PsA, psoriatic arthritis; type I, patients with PsA with type I psoriasis; type II, patients with PsA with type II psoriasis.

*Comparison of phenotype (carriage of one or two alleles) with population controls.

Data shown in n (%) unless stated otherwise.

As expected, the SE was significantly associated with UIA compared with controls (OR 1.88, 95% CI 1.53, 2.29, p = 3.63×10^−10^; table 4 and fig 2). When comparing the UIA and PsA subgroups directly, the frequency of the SE phenotype was significantly higher in UIA subjects compared with those patients with PsA with type I psoriasis (OR 2.08, 95% CI 1.64, 2.66, p = 7.1×10^−10^), but the effect size was lower when comparing UIA subjects to patients with PsA with type II psoriasis (OR 1.51, 95% CI 1.02, 2.22, p = 0.03).

## DISCUSSION

In this large association study of patients with PsA, we have shown that both HLA-Cw*06 and HLA-DRB1*07 are associated with PsA susceptibility in the subgroup of patients with PsA with type I psoriasis but not in those with type II psoriasis, suggesting that the primary association is with type I psoriasis. Our data also confirms that the association with the HLA-DRB1*07 is because this allele is in LD with HLA-Cw*06. We can find no evidence for association of the SE with PsA susceptibility.

The simultaneous investigation of two HLA genes in large cohorts of patients with PsA and appropriate control groups has enabled us to try and dissect out the contribution to PsA over and above that to UIA alone and psoriasis alone. We did not investigate HLA-Cw*06 in UIA patients or *HLA-DRB1* in patients with psoriasis as previous studies have excluded a role for these genes in the respective conditions.^12^ ^30^^–^33 Previous studies on patients with psoriasis have shown that both HLA-Cw*06 and HLA-DRB1*07 are associated with type I psoriasis, but not with type II psoriasis.^12^ ^30^ ^31^ ^42^ We have confirmed that, although both phenotypes show association, the HLA-DRB1*07 result has arisen due to LD with HLA-Cw*06. Furthermore, analysis of HLA-Cw*06 phenotype in patients with type I psoriasis, patients with PsA (stratified according to their types of psoriasis) as well as population controls has allowed us to conclude that the association is primarily with type I psoriasis rather than PsA itself.

Unsurprisingly, HLA-DRB1*04 was found to occur more frequently in the UIA cohort, the majority of whom satisfied American College of Rheumatology classification criteria for RA by 5 years. In contrast, it occurred less commonly in those patients with PsA who were negative for RF and those patients with PsA with type I psoriasis compared with controls. When HLA-DRB1*04 was present in patients with PsA, it occurred more frequently in those with type II psoriasis compared with patients with PsA with type I psoriasis. This may suggest that the HLA-DRB1*04 allele is protective for type I PsA. Alternatively, it may simply reflect the fact that if one allele is increased in frequency (HLA-DR*07 allele frequency increased in patients with PsA with type I psoriasis) then the frequency of others must be reduced.

The broad inclusion criteria for PsA used in this study may have led to the misclassification of some patients who have true RA and coincidental psoriasis being classified as PsA. In this situation, one may have expected to see an association with HLA-DRB1*04 in either the RF-positive subgroup of patients with PsA or those with polyarticular disease but we did not find that to be the case. An advantage of not excluding RF-positive patients is that we have been able to stratify the patients with PsA by their RF status to explore whether RF is an important co-factor in PsA susceptibility. However, in no situation did this stratification change the conclusions of an analysis, suggesting that it is not. Our genetic findings, therefore, accord with recent clinical data suggesting that polyarticular PsA is more similar to oligoarticular PsA than to RA.^43^

We have also confirmed the finding of others that SE was not associated with PsA susceptibility.^28^ ^29^ Unsurprisingly, SE was found to be strongly associated with UIA susceptibility and occurred significantly more frequently in UIA subjects compared with patients with PsA with type I psoriasis. The smaller number of patients with PsA with type II psoriasis may have limited the interpretation of this analysis but the odds of carrying SE was also significantly higher in UIA subjects compared with patients with PsA with type II psoriasis. The findings confirm that, although patients with PsA with type II psoriasis appear more genetically similar to UIA subjects than do patients with PsA with type I psoriasis, UIA and patients with PsA with type II psoriasis are sufficiently different, in terms of their genetic susceptibility, to be viewed as distinct entities.

In summary, our study confirms the established strong association of HLA-Cw*06 with type I psoriasis susceptibility. The association of PsA with HLA-Cw*06 is of similar strength and is confined to those patients with PsA with type I psoriasis. We conclude, therefore, that HLA-Cw*06 does not confer additional susceptibility to IA in patients with psoriasis. We also note that the association between HLA-DRB1*07 and PsA is due to its significant LD with HLA-Cw*06. No independent association was detected with HLA-DRB1*02, HLA-DRB1*04, HLA-DRB1*07 or with the SE and PsA. Our study suggests that the genetic susceptibility of PsA cannot be explained by the HLA-Cw*06 or *HLA-DRB1* loci and confirms the importance of choosing the appropriate control populations when studying this condition. The findings also suggest that adjustment for HLA-Cw*06 is of central importance when attempting to dissect the genetic susceptibility of IA in patients with psoriasis. Finally, our findings suggest that patients with PsA with type I and type II psoriasis have different genetic susceptibility factors and that patients with PsA with type I psoriasis are more genetically similar to type I patients with psoriasis at least at the HLA-Cw*06 locus. This implies that in future genetic studies, it may be important to stratify patients with PsA according to the age of onset of psoriasis.
